# Isolation of ethanol- and acid-resistant *Acinetobacter baumannii* from daqu for efficient esterification of acidic Huangshui

**DOI:** 10.3389/fmicb.2025.1672163

**Published:** 2025-11-24

**Authors:** Jiacheng Li, Yuan Zou, Yumei Jiang, Fei Guo, Lijuan Yang

**Affiliations:** 1 School of Food and Liquor Engineering, Sichuan University of Science & Engineering, Yinbin, China; 2 Brewing Science and Technology Key Laboratory of Sichuan Province, Sichuan University of Science & Engineering, Yibin, China

**Keywords:** Huangshui, esterification ability, *Acinetobacter baumannii*, ethylhexanoate, Huangshui esterified liquid

## Abstract

A large amount of highly acidic wastewater, known as Huangshui, is produced during liquor brewing. To improve its utilization rate and achieve resource recovery of liquor by-products. A strain with high esterifying ability was screened from Daqu and identified as *Acinetobacter baumannii*. The strain exhibited an esterification enzyme activity of (16.79 ± 0.03) U/mL and an esterification capacity of (45.37 ± 0.33) mg/g. It was able to grow normally under conditions of 7% ethanol and pH 4.0, demonstrating strong acid and ethanol tolerance, indicating its potential for preparing a Huangshui esterification solution. Subsequently, Huangshui was esterified using ethanol, caproic acid and Daqu powder prepared with this strain. Analysis of trace components in Huangshui before and after esterification showed that the ethyl caproate content in the esterified Huangshui reached (649.26 ± 2.39) mg/L, which was 13.19 times higher than that before esterification, while ethyl lactate decreased by 15.35%. These results indicate a notable ability to enhance ethyl caproate and reduce ethyl lactate. It is expected that the application of this Huangshui esterification solution could improve the flavor and quality of Non-gxiangxing Baijiu by increasing ethyl caproate and decreasing ethyl lactate, thus providing a reliable experimental basis for the resource utilization of Huangshui.

## 1 Introduction

Liquor-making requires a lot of water, but it will also produce a lot of wastewater. At present, the wastewater produced in liquor brewing is mainly divided into the following five categories: cleaning equipment wastewater, distilled cooling water, distilled bottom pot water, distilled tail water and Huangshui produced during fermentation (Che et al., 2023). Huangshui is a by-product formed by microbial metabolism and free water in raw materials settling at the bottom of pits during the brewing process of Non-gxiangxing Baijiu. It has the characteristics of high organic acid content and high nutrition. If it is discharged directly without treatment, it will be detrimental to comprehensive utilization of resources and environmental protection (Liang et al., 2016). Huangshui mainly contains organic acids, fusel, esters, aldehydes, alcohol, reducing sugar, amino acids, etc., and is rich in microorganisms (Gao et al., 2023). A large number of organic acids in Huangshui endow Huangshui with high acidity, while macromolecular organic substances such as sugar and protein make Huangshui with high biochemical oxygen demand (BOD) and chemical oxygen demand (COD) (Wang et al., 2023). The pH of Huangshui is about 2.5~4.2, the BOD reaches 25,000~30,000 mg/L, and the COD reaches 25,000~40,000 mg/L, which far exceeds the national discharge standard of water pollutants for fermented alcohol and liquor industry (AQSI, 2011). It is precisely due to its high acidity and low pH that the utilization of Huangshui poses a significant challenge (Yao et al., 2009). At the same time, under the current production situation, 300~400 kg of Huangshui is produced for every 1,000 kg of Daqu wine, and 3,000~4,000 t of Huangshui is produced for Daqu wineries with an annual output of 10,000 tons, with a daily output of about 10 t (Yan et al., 2020). The large amount of Huangshui generated not only causes great cost and pressure of sewage treatment for enterprises, but also causes great pollution and waste of resources if it is directly discharged without treatment or improper treatment (Feng et al., 2017). With the development of industry and the continuous expansion of Non-gxiangxing Baijiu industry, the waste of Huangshui resources is a problem that should not be underestimated. For resource protection and ecological environment protection, in addition to improving the process to reduce the generation of waste water, various characteristics of Huangshui can also be used to explore recycling methods (Li, 2012). Huangshui is rich in aroma substances, especially organic acids, but the content of esters which are beneficial to the aroma composition of liquor is relatively little, which makes it have a heavy odor and uncoordinated aroma (Fan et al., 2023). Therefore, many researches focus on increasing the ester content in these by-products of wine-making through esterification, and making mixed liquor with rich ester content. Wu et al. used immobilized enzyme technology to prepare a high-value flavoring liquor with a high concentration of ethyl caproate from a Huangshui esterification solution. This process is relatively simple and has potential for industrial application (Wu et al., 2024). Yang et al. used Huangshui to prepare esterification solution, and then extracted flavor substances by supercritical extraction technology to obtain extract. Adding 3‰ extract to wine can make the wine body relatively soft and harmonious, and improve the quality (Yang et al., 2017).

Esterification of Huangshui is an effective way to achieve rational utilization of Huangshui, improve liquor quality, increase enterprise income, and realize green production. The production of Huangshui esterified liquid involves the use of modern microbial and fermentation engineering technologies to convert organic acids and alcohols in Huangshui into major aromatic compounds in liquor such as esters (Chen et al., 2013; Liu et al., 2020). At present, there are two commonly used esterification methods: microbial enzymatic method and synthetic esterification method. The microbial enzymatic method is widely used in the preparation of Huangshui esterification solution due to its mild reaction conditions, fast reaction rate, high conversion rate, and high product purity (Yu et al., 2021). Xu et al. used self-made Daqu and Monascus to biologically esterify Huangshui, and the liquor obtained was obviously fragrant in grains and esters, with good style and quality (Xu et al., 2020). XIA Q et al. screened a *Monascus purpurea* and added it to Huangshui as an esterifying agent. After continuous reaction for 16 d under the optimum technological conditions, the total ester content in the esterifying solution increased by 1.5 times, and the odor activity values of all esters were improved (Xia et al., 2014).

The characteristics of Huangshui, such as high acidity, high viscosity and high concentration of organic matter, make it difficult for ordinary microorganisms to survive in it (Guo, 2023). In contrast, bacteria have strong resistance to stress, and the contribution of esterification ability of bacteria could not be ignored. Compared to esterifying fungi, esterifying bacteria possess distinct advantages in several aspects, such as faster growth rate (Bornscheuer, 2002), greater environmental adaptability, higher enzymatic activity and stability (Schallmey et al., 2004) broader metabolic diversity, potential for genetic modification (Gao et al., 2019) and lower industrial application costs. These strengths make bacterial esterification highly promising for both biosynthesis and industrial production. In this study, a functional bacterium with high tolerance to ethanol and pH was screened which can be used to prepare a Huangshui esterification solution. This solution has the potential to be applied in the cross-steaming process of Non-gxiangxing Baijiu to improve liquor quality.

## 2 Materials and methods

### 2.1 Materials and reagents

Daqu was provided by a Non-gxiangxing winery in Yibin. It was crushed into powder and packed in clean plastic bags. Yeast powder, peptone, sodium chloride and agar powder were purchased from Beijing Aoboxing Biotechnology Co., Ltd.; Glycerol, anhydrous ethanol and concentrated sulfuric acid were purchased from Chengdu Cologne Chemical Co., Ltd.; Glyceryl tributyrin and α -naphthyl acetate were purchased from Shanghai McLean Biochemical Technology Co., Ltd.; Fast Blue B Salt, α-naphthol and NaOH were purchased from Mulan Town Industrial Development Zone, Xindu District, Chengdu. Sodium dodecyl sulfate (SDS) was purchased from Chengdu Kelong Chemical Reagent Factory. Amyl acetate (Standard for GC,99.5%), Internal standard for GC-MS analysis, Purchased from Shanghai McLyn Biochemical Technology Co., Ltd..

### 2.2 Culture conditions

LB medium: peptone 10 g/L, yeast powder 5 g/L, NaCl 10 g/L, pH 7.0; To prepare the solid medium, 20 g/L AGAR is added. Initial screening medium: yeast powder 3 g/L, peptone 10 g/L, NaCl 5g/L, tributyrin 0.4%, pH 7.0~7.2. Basic fermentation medium: 30 g bran, 30 mL distilled water.

The sterilization conditions of the above medium were 121 °C for 20 min.

### 2.3 Strain screening

(1) Preliminary screening

Weigh 25 g of crushed Daqu, add it to 250 mL of LB liquid medium, enrich and cultivate it for 24 h at 37 °C and 180 r/min, dilute 1 mL of enriched liquid to 9 mL of sterile physiological saline to 10^−9^ according to a gradient of 10 times, and then spread 100 μL of diluted liquid on LB solid medium, and cultivate it for 24 h at 37 °C. Different colonies were selected from the plate and separated and purified for many times until they were single colonies on the plate culture medium. The isolated and purified strain was inoculated into LB liquid culture medium for activation, and cultured at 37 °C and 180 r/min for 24 h, which was the crude enzyme preparation. Put three Oxford cups in the primary screening medium, take 100 μL of crude enzyme preparation in the Oxford cups, and culture at 37 °C for 24 h. Measure the transparent circle directly (D) and the colony diameter (D) with the bacteriostatic circle measuring instrument, and calculate the D/d value. Select strains with high D/d value for further re-screening. The screened strains were numbered and stored in a 50% glycerol tube, and stored in a refrigerator at−20 °C for later use.

(2) Re-screening

Inoculate the strains obtained by primary screening into LB liquid culture medium for activation, culture at 37 °C and 180 r/min for 24 h, take 5 mL of bacterial liquid and inoculate it into the basic fermentation medium, stand at 30 °C for 7 d, The cultured *qu* sample was spread evenly on shallow trays and dried in an oven at 40 °C. After 24 h, the dried sample was ground into powder and stored at 4 °C. The viable bacterial count of the resulting *qu* powder was approximately 1 × 10^8^ – 10^10^ CFU/g. Another 50 mL of bacterial liquid was centrifuged at 4 °C and 12,000 rpm for 10 min, and the supernatant was collected as crude enzyme liquid. The crude enzyme activity was determined by colorimetry and the esterification power was determined by titration. The enzyme activity of the strain was analyzed according to the results of enzyme activity determination, and the ester-producing ability and specificity of the strain were analyzed according to the esterification power and gas chromatography.

### 2.4 Esterase activity determination

The esterase activity was determined by α-naphthol colorimetry (Liu et al., 2019):α-naphthalene acetate is hydrolyzed to α-naphthalene ester and acetic acid by esterification enzyme at 37 °C and pH 7.0, and α-naphthalene ester has a color reaction with Fast Blue B Salt to produce a diamond-red compound. The absorbance of the compound can be measured at 528 nm wavelength, and the size of the absorbance reflects the amount of α-naphthyl ester, and then the enzyme activity of esterase can be calculated.

0.5 mL supernatant was mixed with 3 mL phosphoric acid buffer at pH 6.0, 0.1 mL naphthalene acetate was added, the blank group was not added, and 0.4 mL solid blue B salt was added after reaction in water bath at 37 °C for 15 min, and the absorbance was measured at wavelength 528 nm at 37 °C for 10 min.

Esterase activity definition: at 37 °C and pH 7.0, the required enzyme amount of 1 enzyme activity unit (U/mL) for hydrolysis of α-naphthol acetate for 15 min to produce 1.0 nmol α-naphthol.

### 2.5 Determination of esterification ability

Take 1.5 mL caproic acid, add 25 mL ethanol, shake well, then add 75 mL pure water, shake well, then add 5 g fermented yeast powder of the strain, shake well, tighten with a sealing film, make esterification solution, and put it in a 35 °C incubator for esterification for 100 h, and then test the esterification ability. Refer to light industry standard QB/T 4257-2011 “General Analytical Method for Distillery Daqu” for specific determination and operation methods (MIIT, 2011).

The unit definition of esterification ability: the number of milligrams of organic acid and ethanol synthesized by organic acid ethyl ester catalyzed by 1 g dry yeast at a certain temperature for a certain time is a unit, expressed in mg/g.

### 2.6 Analysis of synthesis ability of four esters by GC

Suck 0.375 mL caproic acid, 0.375 mL acetic acid, 0.375 mL lactic acid and 0.375 mL butyric acid into a 250 mL conical flask, add 25 mL absolute ethanol and 75 mL distilled water, and mix thoroughly. Weigh 5 g of the dried sample, add it into the conical flask, shake it evenly, seal it with sealing film, put it in a constant temperature incubator at 35 °C for 100 h, and do a blank experiment at the same time, and then distill it to get the distillate. Take 940 μL distillate and add 60 μL internal standard for GC analysis (Zhang et al., 2023; Zhou et al., 2023). The gas chromatography standard curves for each ester class can be found in the supplement.

### 2.7 Identification of strains

Morphological identification (Moyes et al., 2009): Morphological observation: The re-screened strains were diluted on LB solid medium and cultured at 37 °C for 12 h. The size, shape, color and eminence of the colony were observed by naked eye. Microscopic morphology observation: The color and shape of the strain were observed under an oil mirror after gram staining.

Contact enzyme test: Drop a drop of 3% hydrogen peroxide solution on the slide, select a little of the culture of the screened bacteria colony on the reagent, observe whether there are bubbles, if there are bubbles, it is positive, and no bubbles are negative.

Molecular biological identification: DNA of the re-screened strain was extracted, and it was used as a template for PCR amplification with universal bacterial primers (27f:5′ AGAGTTTGATCCTGGCTCAG3′ and 1492r:5′ GGTTACCTTGTTAGGACTT3′) (Zhang et al., 2020). The PCR products were detected by 1% agarose gel electrophoresis and sent to Beijing Qingke Biotechnology Co., Ltd. for sequencing. The sequencing results were submitted to the GenBank database of National Biotechnology Information Center (NCBI) for Blast comparison, and the phylogenetic tree was constructed by the “N-J neighborhood method” in MEGA 11 (Hall, 2013).

### 2.8 Analysis of strain characteristics

Ethanol tolerance determination (Lai et al., 2022): 0%, 1%~13% anhydrous ethanol was added to LB liquid medium, and the screened strains were inoculated in the medium at 10% inoculum volume, and cultured at 37 °C and 180 r/min for 24 h. The absorbance was measured at 600 nm wavelength with the base zero of unconnected bacteria culture.

Acid resistance determination (Moradi et al., 2018): In LB liquid medium, the pH of the medium was adjusted from 1 to 9, and the selected strains were inoculated in the medium at 10% inoculation rate, and cultured at 37 °C and 180 r/min for 24 h. The absorbance was measured at 600 nm wavelength with the base zero of unconnected bacteria culture.

### 2.9 Preparation and analysis of Huangshui esterification solution

Fresh Huangshui from a brewery was taken, and the upper suspended matter and lower sediment were removed after standing for 1 week. The total ester, total acid, alcohol content and pH of the treated Huangshui were determined and analyzed by gas chromatography-mass spectrometry. Amyl acetate was used as an internal standard for quantification at a concentration of 2.0404 g/100 mL. Preparation of Huangshui esterification solution: measure 10 mL of ethanol in a conical flask, add 1 mL of caproic acid and 90 mL of Huangshui (the pH is adjusted to 4.0), then add 5 g of the dried sample of the re-screened strain, and esterify in a constant temperature incubator at 35 °C for 7 days. The esterification power of Huangshui esterification solution was determined and the main trace components were analyzed by chromatography.

Determination of total ester: refer to QB/T 4257-2011 “General Analytical Method for Distillery Daqu” (MIIT, 2011).

Determination of total acid: refer to GB/T 12456-2008 “Determination of Total Acid in food” (NHC and S. A. M. R., 2021).

Determination of alcohol: Direct determination with alcohol meter.

Determination of pH: Direct determination of pH meter.

Analysis by GC-MS (Fan and Qian, 2005): GC conditions: DB-WAX (30 m × 0.25 mm × 0.25 m) column; The carrier gas is helium (99.999%), the constant flow rate is 1 mL/min, the injection mode is not split, and the injection temperature is 250 °C. Heating procedure: the initial temperature is 40 °C for 3 min, and the temperature is raised to 230 °C at 5 °C/min, and kept for 7 min. MS conditions: ion source inlet temperature 250 °C; Heating program: the initial temperature is 40 °C for 3 min, and the temperature is raised to 230 °C at 5 °C/min for 7 min. The (EI) temperature is 230 °C and the electron energy is 70 eV;. Mass scanning range m/z 35 ~ 350. Sample volume: 1 μL.

### 2.10 Data processing

There were three replicates of the experiment in this study. Data processing is performed using software such as Excel 2016 and Origin 2018 for statistics and plotting.

## 3 Results and discussion

### 3.1 Screening of strains

Twelve strains of bacteria were isolated and purified from Daqu These strains were screened using a primary screening medium containing tributyrin, and the results are shown in Table 1. The D/d values of the 12 strains ranged from 1.57 to 3.35, indicating that all of them had the ability to hydrolyze tributyrin. Strain D-H exhibited the largest D/d value. The composition of the primary screening medium includes a small amount of tributyrin, and some strains secrete esterifying enzyme, which has the ability to decompose tributyrin, and the transparent circle around the colony represents the decomposed tributyrin. Therefore, the larger the ratio of transparent circle diameter to colony diameter, the better the esterifying enzyme produced by the strain (Liu, 2013). Six strains with large D/d values (D-H, D-W, D-Y, Z-2, Z-8, D) were selected for further study.

**Table 1:** Rations of transparent circle diameter(D) and colony diameter(d) of primary creened strains.

**Strain**	**D/*d* value**	**strain**	**D/*d* value**
D-H	3.35	Z-9	1.80
D-W	2.71	Q-2	1.65
D-Y	2.65	D-11	1.63
Z-2	2.03	Q-3	1.58
Z-8	1.98	Z-1	1.57
D-2	1.81	Z-5	1.57

**Figure 1:**
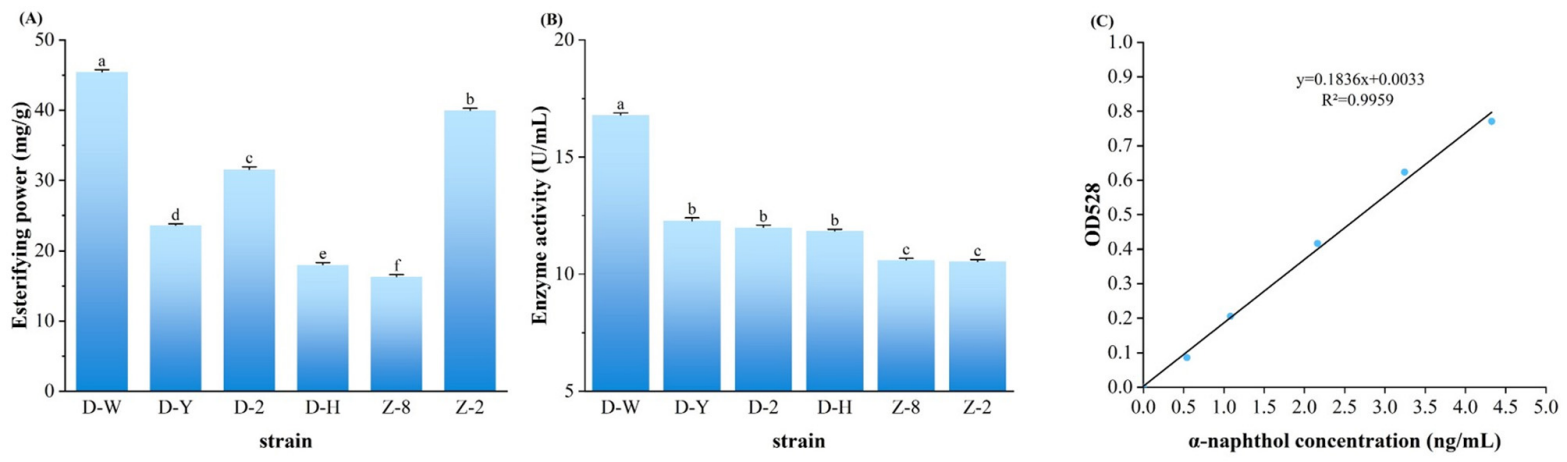
Rescreening and identification of the six microbial strains. **(A)** Esterase hydrolytic activity of each strain determined by the a-naphthol assay. **(B)** Esterification synthesis capability of each strain, represented by ethyl caproate production, as determined by gas chromatography (GC) analysis. **(C)** a-Naphthol standard curve for the quantification of esterase activity. different lowercase letters indicate statistically significant differences (*P* < 0.05) as determined by one-way ANOVA with a *post-hoc* test.

The esterase activity of 6 strains selected for re-screening (D-H, D-W, D-Y, Z-2, Z-8, D-2) was determined as shown in Figure 1A, while the esterification power was determined as shown in Figure 1B, the α-naphthol solution standard curve was determined as shown in Figure 1C. The highest enzyme activity of D-W could reach (16.79 ± 0.03) U/mL, showing strong enzyme production ability. The six strains D-W, D-Y, D-2, D-H, Z-8 and Z-2 had higher enzyme activity and could be screened in the next step. As can be seen from Figure 1B, the above strains D-W, Z-2 and D-2 all had the ability to synthesize esters, among which strain D-W had the highest esterification power, which was (45.37 ± 0.33) mg/g, higher than the 30 mg/g specified in the enterprise standard QB/T5188-2017 “Brewing Red Yeast”(MIIT, 2017). Esterification enzyme has the function of decomposing ester and synthesizing ester at the same time. Enzyme activity measurement reflects the ability of decomposing ester, while esterification ability measurement reflects the ability of synthesizing ester (Zhang and Huang, 2016). According to the determination results of esterifying enzyme activity and esterifying power, D-W in the re-screened strain has a strong ability to produce esterase, indicating that it is the target strain of this study.

As can be seen from Table 2, strain D-W had the strongest ability to synthesize ethyl caproate, and the synthesis amount was as high as (778.18 ± 0.13) mg/L. D-2 also had the ability to synthesize ethyl caproate, but it was weaker than D-W. Compared with the blank control, the contents of ethyl caproate, ethyl acetate, ethyl butyrate and ethyl lactate were reduced after esterification by Z-8, Z-2, D-H and D-Y strains, indicating that these strains had the ability to decompose ester. For D-W and D-2 strains, after esterification, the amount of ethyl caproate was significantly increased, and the content of ethyl acetate was significantly decreased, indicating that these two strains may have the ability to “increase hexyl and reduce ethyl”. Since the main purpose of this study was to produce ethyl caproate, D-W was selected as the target strain for strain identification according to Figure 1 and Table 2.

**Table 2:** Detection of synthesis ability of four esters of strain.

**Strain**	**Ethyl acetate**	**Ethyl butyrate**	**Ethyl caproate**	**Ethyl lactate**
Blank group	424.23 ± 0.06	196.45 ± 0.06	133.13 ± 0.07	73.53 ± 0.07
D-W	81.63 ± 0.12^***^	160.15 ± 0.12^***^	778.18 ± 0.13^***^	80.04 ± 0.06^***^
Z-8	96.30 ± 0.10^***^	66.66 ± 0.05^***^	54.41 ± 0.06^***^	ND
Z-2	74.16 ± 0.11^***^	66.74 ± 0.07^***^	82.01 ± 0.08^***^	ND
D-H	69.73 ± 0.04^***^	44.16 ± 0.08^***^	11.54 ± 0.08^***^	ND
D-Y	70.42 ± 0.05^***^	45.84 ± 0.06^***^	16.27 ± 0.01^***^	ND
D-2	72.83 ± 0.05^***^	81.43 ± 0.06^***^	295.07 ± 0.04^***^	56.12 ± 0.05^***^

The blank group is the esterified solution sample without adding strain koji powder; “ND” indicates no detection. ^***^ indicates a highly significant difference from the blank group (*P* < 0.01).

### 3.2 Identification of strains

**Figure 2:**
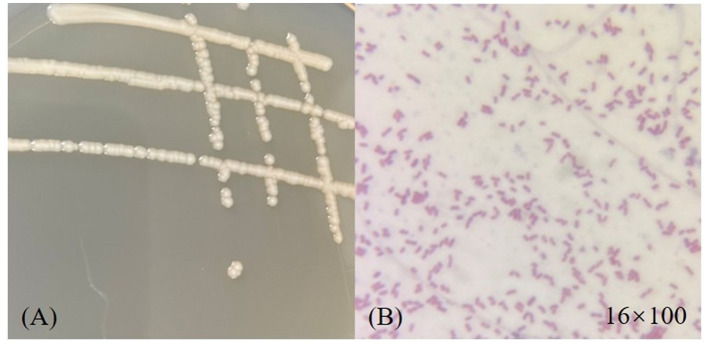
Morphological characterization of strain D-W. **(A)** Colonial morphology on an LB agar plate, showing round, smooth, moist, gray- white colonies of medium size. **(B)** Microscopic view under oil immersion (1600x magnification) after Gram staining, revealing red-colored, rod- shaped cells, indicative of a Gram-negative bacterium.

**Figure 3:**
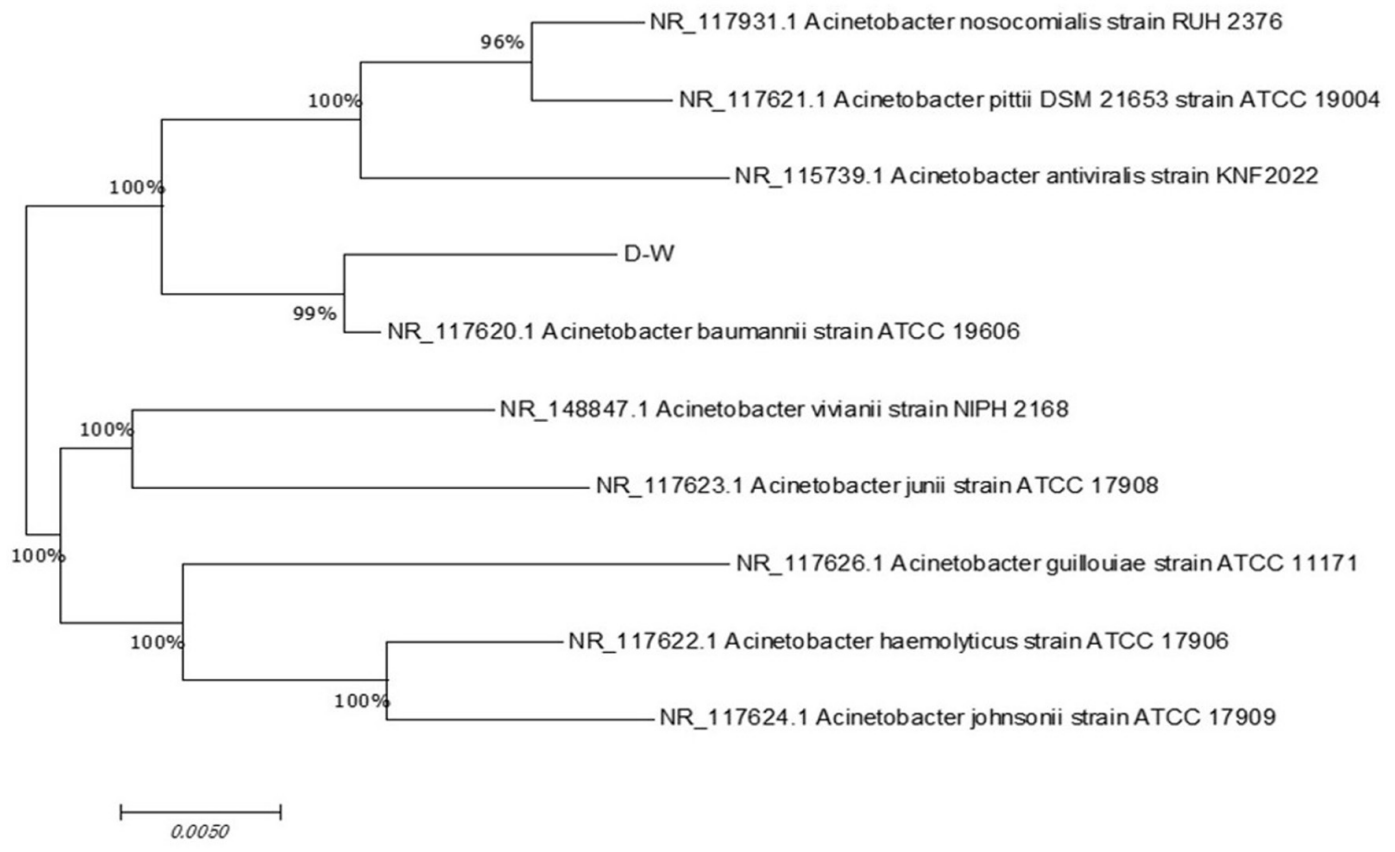
Phylogenetic analysis of strain D-W based on 16S rRNA gene sequence. The evolutionary tree was constructed using the neighbor-joining method and illustrates the phylogenetic relationship between strain D-W and closely related type strains of the genus Acinetobacter. Bootstrap values (percentage, shown at the nodes) were used to assess the reliability of the branches. The scale bar (0.0050) indicates the number of nucleotide substitutions per site. Strain D-W exhibits the highest sequence similarity (99%) with Acinetobacter baumannii, confirming its classification within this species.

The morphology of strain D-W was observed visually: after 24 h of culture on LB solid medium at 37 °C, colonies appeared as smooth, moist, ivory-colored round dots with neat edges and a slightly convex profile. The microscopic picture of the strain is shown in Figure 2. under the optical microscope, it is mostly club-shaped. Gram staining revealed that strain D-W is a Gram-negative bacterium. The enzyme contact test was positive. Combined with its colonial morphology, the strain was preliminarily identified as Gram-negative.the 16srDNA sequence of the isolate was submitted to GenBank and compared with homologous sequences. Phylogenetic analysis was performed using MEGA11 software, and the phylogenetic tree of strain D-W is shown in Figure 3. Strain D-W showed a very close phylogenetic relationship with *Acinetobacter baumannii*, with a sequence similarity of 99%. Based on morphological, physiological, and biochemical characteristics, strain D-W was preliminarily identified as *Acinetobacter baumannii*. The strain has been deposited in the China Center for Type Culture Collection (Wuhan, China) under accession number CCTCC NO: M 20231418.

### 3.3 Analysis of strain characteristics

**Figure 4:**
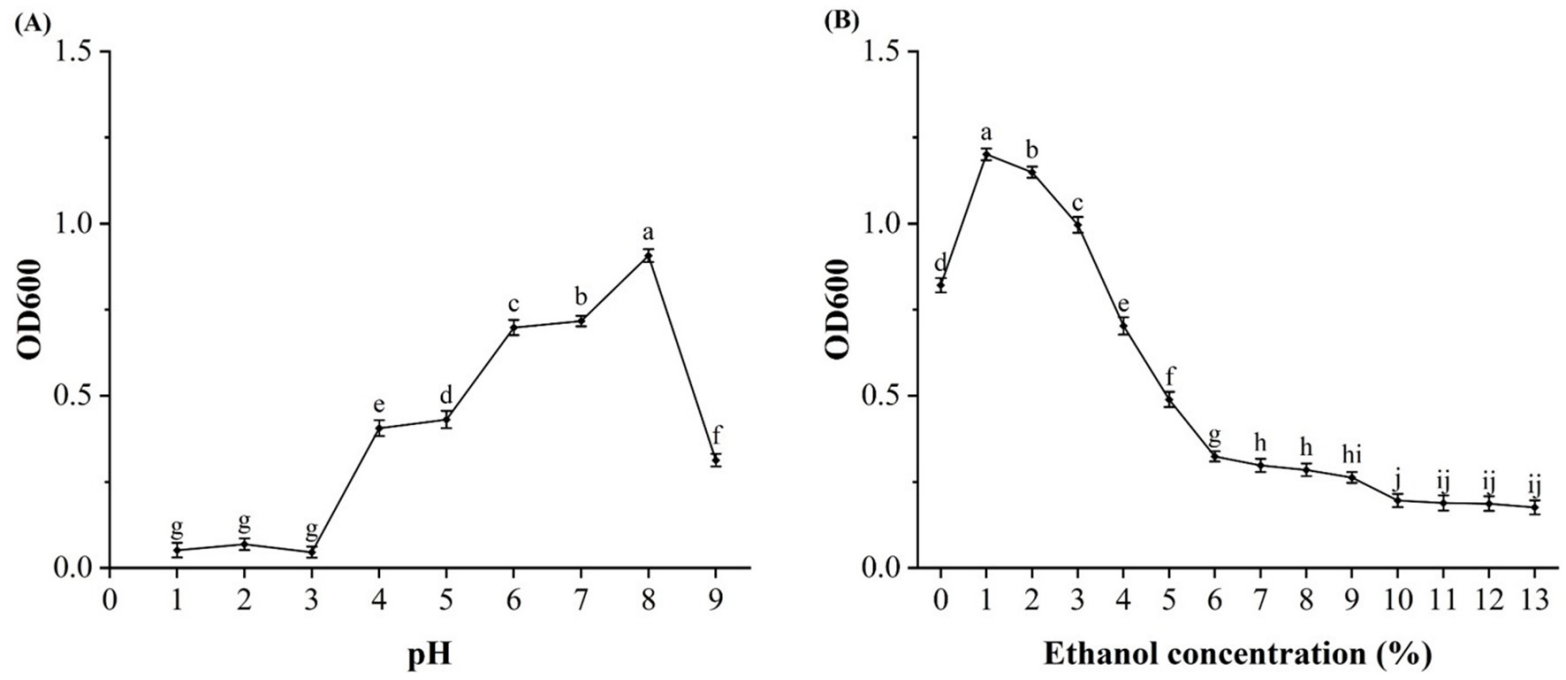
Evaluation of pH and ethanol tolerance in strain D-W. **(A**) Growth of strain D-W under different initial pH levels. **(B)** Growth of strain D-W in the presence of different ethanol concentrations. Data are shown as mean + SD; different lowercase letters above bars indicate statistically significant differences (*P* < 0.05) as determined by one-way ANOVA with a *post-hoc* test. The results define the optimal and limiting growth conditions for the strain.

The tolerance of D-W strain to ethanol and pH was investigated, and it was expected that the strain could be used in Huangshui (because Huangshui is rich in organic acids and alcohols). According to Figure 4A, strain D-W could grow normally at pH 4~9, and pH 8 was the optimal growth concentration, at pH 4, the strain can still grow. Based on Figure 4B, strain D-W could grow normally at an alcohol concentration of 0% to 7%, and 1% alcohol was the optimal growth concentration, when the alcohol concentration is 9%, the strain can still grow, indicating that D-W had relatively strong acid and ethanol resistance, and can survive in the environment of high acidity, high viscosity and high organic content of Huangshui, and has the potential to prepare Huangshui esterification solution.

### 3.4 Analysis of Huangshui composition and esterification solution of Huangshui

The results of routine analysis and GC-MS analysis of Huangshui are shown in Table 3 and Table 4. Huangshui is rich in alcohols, acids and esters. Notably the contents of L- lactic acid, caproic acid and ethanol are particularly high, reaching 23,126.76 mg/L, 5,525.86 mg/L and 22,507.37 mg/L respectively. Although Huangshui contains a variety of esters, the content of ethyl caproate is relatively low at only 22.04 mg/L, while ethyl lactate is the most abundant ester, with a content of 930.58 mg/L. The high concentrations of organic acids and alcohols suggest that Huangshui has significant potential for microbial esterification.

**Table 3:** The routine analysis of Huangshui.

**Item**	**Alcoholic strength (%, V/V)**	**Total acid (g/100mL)**	**Total ester (mg/100mL)**	**pH**
Content	ND	4.47 ± 0.096	533.49 ± 10.034	3.32 ± 0.625

“ND” indicates no detection.

**Table 4:** The contents of main trace components of Huangshui.

**Matter**	**Concentration (mg/L)**
Ethyl oxamate	16.47 ± 0.04
Ethyl acetate	104.25 ± 0.03
Ethyl caproate	22.04 ± 0.03
Ethyl lactate	930.58 ± 0.12
L-lactic acid	23126.76 ± 5.24
Acetic acid	4502.56 ± 1.23
Butyric acid	1328.83 ± 1.13
Caproic acid	5525.86 ± 2.34
(R)-1, 2-propylene glycol	1333.57 ± 1.02
Glycerin	15399.10 ± 4.23
Ethyl alcohol	22507.37 ± 5.67
Propyl alcohol	71.29 ± 0.07
N-butanol	112.24 ± 0.12
N-amyl alcohol	16.29 ± 0.02
Acetone alcohol	18.72 ± 0.05
N-hexyl alcohol	71.98 ± 0.11
(2R,3R)-(-)-2, 3-butanediol	491.47 ± 0.32
2, 3-butanediol	440.51 ± 0.27
Propanediol	2279.99 ± 1.34
1, 3-propylene glycol	105.97 ± 0.39
Phenylethanol	129.89 ± 0.26
1, 6-dextropyranose	20.87 ± 0.08
Dihydroxymaleic acid	19.66 ± 0.03

**Figure 5:**
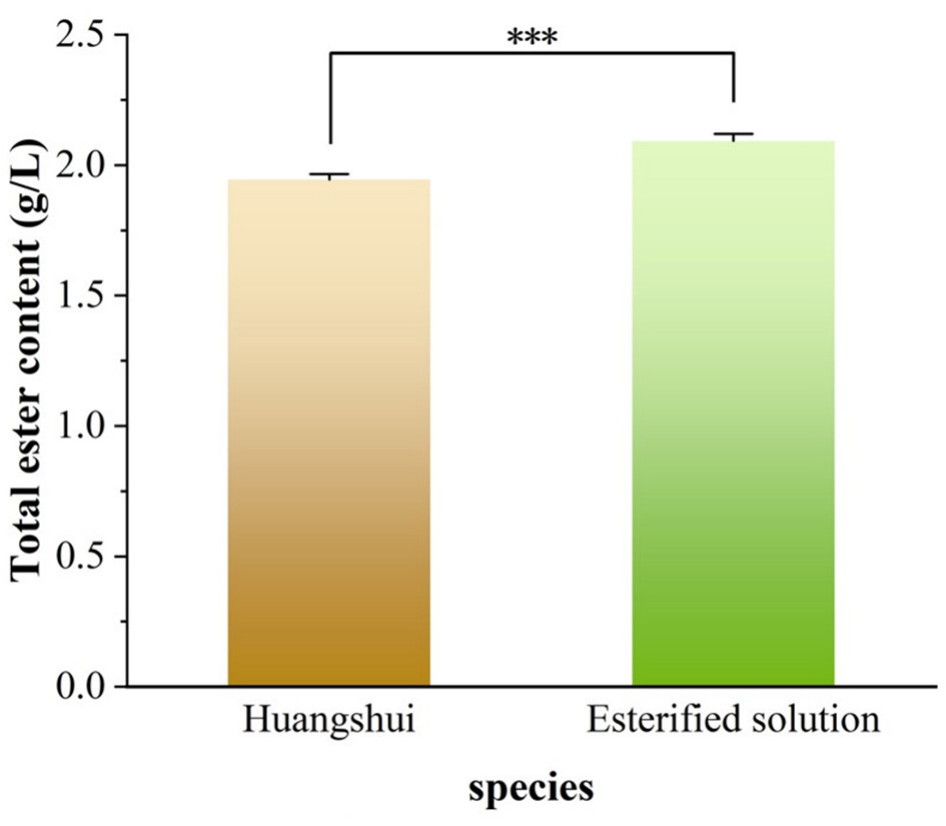
Determination of total ester content in esterification systems. The total ester content was measured to evaluate the esterification capability. A highly significant difference (^***^, *P* < 0.001) between the “Huangshui” (control) and “Esterified solution” (test) groups demonstrates a substantial enhancement in ester synthesis due to the catalytic process.

**Figure 6:**
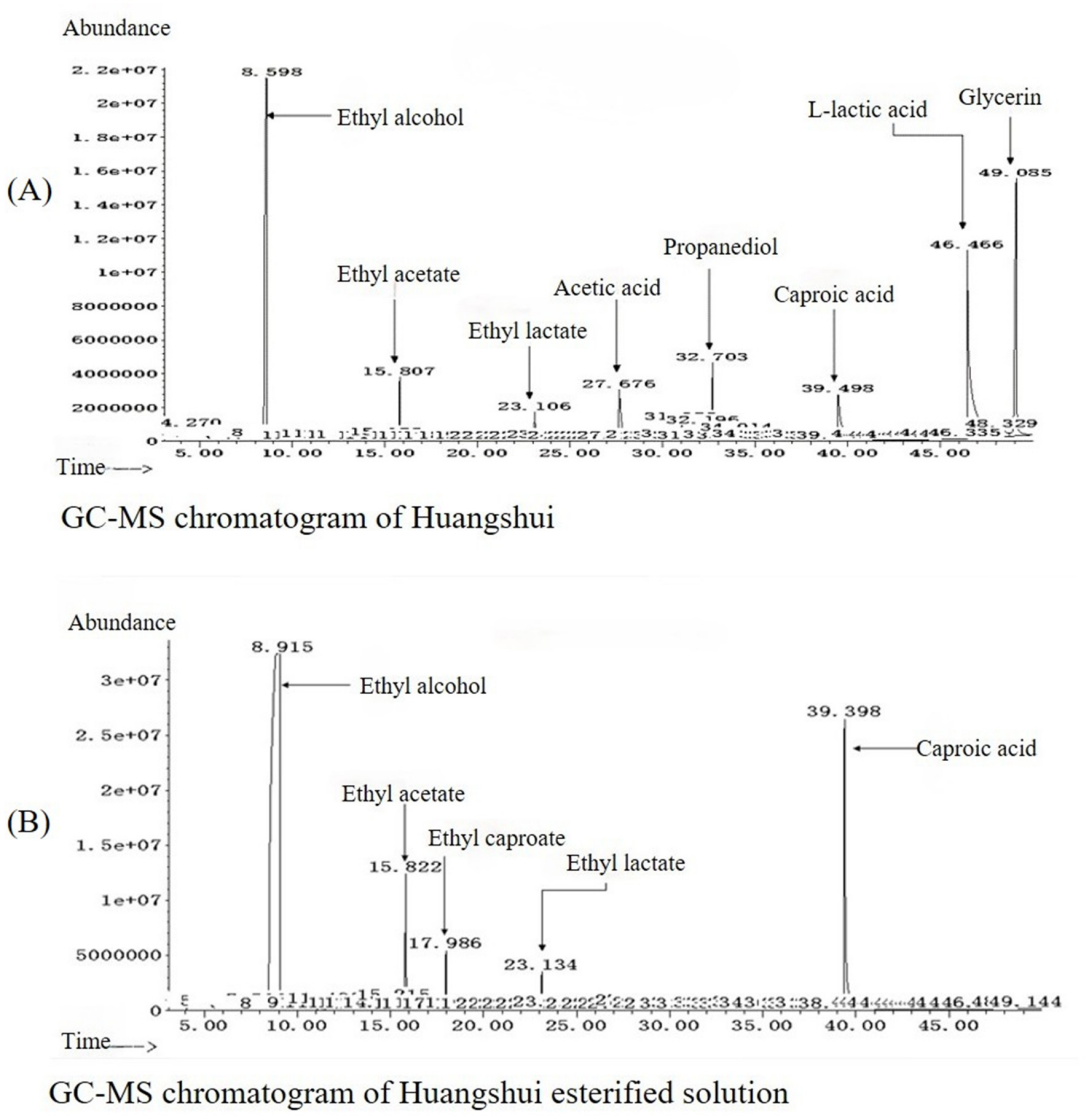
Comparative GC-MS chromatograms of volatile compounds in the sample before and after esterification. **(A)** Chromatogram of the control group (raw Huangshui), in which components such as ethyl alcohol, L-lactic acid, glycerin, and acetic acid were identified. **(B**) Chromatogram of the esterified sample, revealing a substantial compositional shift. Notable changes include a significant increase in ethyl acetate and the appearance of newly detected ethyl caproate, whereas 39.398 components like L-lactic acid and glycerin were substantially reduced or absent.

As can be seen from Figure 5, the total ester content in the Huangshui esterified solution prepared using the fermented Fuqu powder inoculated with Acinetobacter baumannii increased by approximately 8% compared with the non-esterified Huangshui indicating that the strain can promote the synthesis of ester substances to some extent. The GC-MS chromatogram of Huangshui and Huangshui esterified solution is shown in Figure 6. Further analysis of trace components in the esterified Huangshui (Table 5) revealed that ethyl caproate—a key flavor compound in Non-gxiangxing Baijiu—increased from 49.21 mg/L to 649.26 mg/L after esterification, making it the dominant flavor component in the solution. Ethyl valerate rose from 0 mg/L to 7.54 mg/L, and caproic acid increased from 5,224.70 mg/L to 5,751.67 mg/L. In contrast, acidic substances such as acetic acid, isobutyric acid, and 6-bromocaproic acid decreased, as did fusel oils including n-hexanol, propylene glycol, and phenylethanol. Ethyl lactate and ethyl butyrate also declined to varying degrees. These shifts suggest that Acinetobacter baumannii may facilitate “increasing ethyl caproate while decreasing ethyl lactate” and “increasing ethyl caproate while decreasing ethyl acetate” (Xu, 2021).

**Table 5:** The contents of main trace components of Huangshuir esterification solution.

**Matter**	**Concentration (mg/L)**	
	**Huangshui**	**Huangshui esterification solution**
Amyl acetate	1540.80 ± 1.24	1540.80 ± 1.25
Ethyl caproate	49.21 ± 0.03	649.26 ± 2.39^***^
Ethyl acetate	52.59 ± 0.04	42.67 ± 0.09^***^
Ethyl lactate	630.68 ± 0.43	533.84 ± 3.36^***^
Ethyl butyrate	7.07 ± 0.02	6.77 ± 0.01^**^
Ethyl valerate	ND	7.54 ± 0.01^***^
Dimethyl carbonate	175.92 ± 0.56	33.72 ± 0.04^***^
Ethyl formate	1.68 ± 0.03	1.06 ± 0.01^***^
Acetic acid	285.79 ± 0.17	220.31 ±0.33^***^
Isobutyric acid	6.72 ± 0.02	ND
Butyric acid	142.14 ± 0.11	120.44 ± 0.12^***^
6-bromocaproic acid	63.69 ± 0.61	ND
N-valeric acid	26.72 ± 0.02	63.31 ± 0.09^***^
Caproic acid	5224.70 ± 5.98	5751.67 ± 4.08^***^
Isoamyl alcohol	14.97 ± 0.03	14.00 ± 0.03^***^
N-amyl alcohol	7.57 ± 0.01	7.75 ± 0.01
N-hexyl alcohol	19.70 ± 0.03	19.29 ± 0.02^**^
Propanediol	34.65 ± 0.03	13.14 ± 0.02^***^
Phenylethanol	103.10 ± 0.07	35.57 ± 0.34^***^
Ethyl alcohol	38435.11 ± 3.89	38238.81 ± 6.15^***^
Propyl alcohol	61.58 ± 0.12	9.88 ± 0.23^***^
N-butanol	79.66 ± 0.12	78.62 ± 0.56^***^
Acetaldehyde	21.09 ± 0.02	16.51 ± 0.04^***^

Amyl acetate in the table is the internal standard added during GC-MS analysis; “ND” indicates no detection. ^**^Significantly different from the blank group (*P* < 0.05), ^***^ indicates an extremely significant difference from the blank group (*P* < 0.01).

The observed changes are likely attributable to the metabolic activity of the strain. This is consistent with previous studies: for example, Han et al. (2019) isolated an *Acinetobacter johnsonii* strain from Songhe pit mud that showed high esterase production (activity of 2.83 U/mL). Similarly, Zhang et al. (2022) performed whole-genome sequencing on *Acinetobacter* sp. WCO-9, a high-lipase-producing strain, confirming that *Acinetobacter* species are an important source of microbial lipases. These reports indicate that bacteria within this genus commonly possess esterase/lipase activity. Together with the positive esterase activity observed during our initial screening, it is reasonable to conclude that the altered ester profile resulted from the metabolic function of the *A. baumannii* strain used in this study.

Table 5 also shows that acetic acid decreased from 285.79 mg/L to 220.31 mg/L, butyric acid decreased from 142.14 mg/L to 120.44 mg/L, while caproic acid increased by approximately 10%. This suggests that Acinetobacter baumannii may possess the ability to convert acidic substances such as acetic acid and butyric acid into caproic acid, thereby promoting the production of ethyl caproate and the consumption of other acids in the Huangshui esterification solution. After distillation, the esterified solution can be used as a flavor liquor to help modulate the ethyl caproate content in Non-gxiangxing Baijiu. Studies have indicated that Acinetobacter baumannii can produce various synthetically active esterases/lipases, which are capable of catalyzing ester synthesis or transesterification reactions in non-aqueous or low-water phase systems *in vitro*, demonstrating catalytic versatility beyond their innate hydrolytic activity (Li et al., 2010).

## 4 Conclusions

Twelve esterase-producing strains were isolated from Daqu, Among them, strain D-W exhibited the highest enzyme activity of (16.79 ± 0.03) U/mL, along with the strongest capacity for ethyl caproate synthesis, measured at (45.37 ± 0.33) mg/g. Based on morphological, physiological, biochemical, and 16S rDNA phylogenetic analyses, the strain was preliminarily identified as *Acinetobacter baumannii*. Historically, research on esterifying microorganisms has primarily focused on molds and yeasts, yet the contribution of bacteria should not be overlooked. The *A. baumannii* strain screened in this study not only enriches the microbial resources for bacterial ester production but also provides a reference for the application of esterifying bacteria.

The strain demonstrated normal growth under conditions of pH 4 and 7% ethanol, indicating strong stress tolerance. In a fermentation system consisting of 90 mL of Huangshui, 1 mL of caproic acid, and 10 mL of anhydrous ethanol, GC-MS analysis revealed that the ethyl caproate content increased by 13.19-fold compared to the blank control. This suggests that the fermented product has potential for enhancing the aroma profile of Non-gxiangxing Baijiu. When applied in the preparation of Huangshui esterification solution, the strain effectively reduced the levels of fusel oils and other acidic substances while increasing the content of the desired aroma component—ethyl caproate—thereby significantly improving the utilization efficiency of Huangshui. To achieve optimal results, further optimization of the esterification process parameters such as the amounts of ethanol and caproic acid, esterification time, and temperature is recommended.

## Data Availability

The original contributions presented in the study are included in the article/Supplementary material, further inquiries can be directed to the corresponding author/s.
