# Supplementary material for: Isolation of ethanol- and acid-resistant Acinetobacter baumannii from daqu for efficient esterification of acidic Huangshui
Source: Front Microbiol. 2025 Nov 24;16:1672163. doi: 10.3389/fmicb.2025.1672163 (PMC12683909; doi:10.3389/fmicb.2025.1672163)
Supplement: Supplementary file 1 [file Table_1.DOCX]

**Table standard curve of gas chromatography**

| Matter | Peak time | R² | RSD% | Formula |
| --- | --- | --- | --- | --- |
| Ethyl acetate | 4.677 | 99.67% | 2.75% | y = 3.0832x + 0.0484 |
| Ethyl butyrate | 9.571 | 99.66% | 2.78% | y = 1.9106x + 0.0277 |
| Ethyl hexanoate | 19.696 | 99.95% | 2.37% | y = 1.4151x + 0.0015 |
| Ethyl lactate | 23.645 | 99.83% | 3.56% | y = 1.9123x + 0.0289 |

The concentrations of the four major esters in the esterification solution were calculated based on the gas chromatography calibration curve, and the results are shown in table 2.
